# Prolyl Isomerase Pin1 in Human Cancer: Function, Mechanism, and Significance

**DOI:** 10.3389/fcell.2020.00168

**Published:** 2020-03-31

**Authors:** Wenchen Pu, Yuanyuan Zheng, Yong Peng

**Affiliations:** Laboratory of Molecular Oncology, State Key Laboratory of Biotherapy and Cancer Center, West China Hospital, Sichuan University Collaborative Innovation Center of Biotherapy, Chengdu, China

**Keywords:** Pin1, PPIase isomerase, phosphorylation, *cis-trans* isomerization, cancer hallmarks

## Abstract

Peptidyl-prolyl *cis-trans* isomerase NIMA-interacting 1 (Pin1) is an evolutionally conserved and unique enzyme that specifically catalyzes the *cis-trans* isomerization of phosphorylated serine/threonine-proline (pSer/Thr-Pro) motif and, subsequently, induces the conformational change of its substrates. Mounting evidence has demonstrated that Pin1 is widely overexpressed and/or overactivated in cancer, exerting a critical influence on tumor initiation and progression via regulation of the biological activity, protein degradation, or nucleus-cytoplasmic distribution of its substrates. Moreover, Pin1 participates in the cancer hallmarks through activating some oncogenes and growth enhancers, or inactivating some tumor suppressors and growth inhibitors, suggesting that Pin1 could be an attractive target for cancer therapy. In this review, we summarize the findings on the dysregulation, mechanisms, and biological functions of Pin1 in cancer cells, and also discuss the significance and potential applications of Pin1 dysregulation in human cancer.

## Introduction

Cellular processes are spatially and temporally regulated by a number of molecular machineries consisting of proteins and nucleic acids (Csizmok et al., 2016; Koelwyn et al., 2017; Hentze et al., 2018). Diverse regulatory mechanisms have been well established to interpret cellular processes, such as epigenetic changes, allosteric regulations, and post-translational modifications (Aebersold and Mann, 2016; Changeux and Christopoulos, 2016; Luo et al., 2018). Among them, post-translational modifications are currently emerging as an important regulator of cell fate and thus have a strong potential to be implicated in cellular disorders (Barber et al., 2018; Steklov et al., 2018). As a dominative component of post-translational modifications, protein phosphorylation in response to extracellular or intracellular stimuli mainly controls the signal transduction within cells (Boss and Im, 2012), which often includes conformational changes in kinase-phosphorylated substrates (He et al., 2015; Martin et al., 2016). Therein, the conformational switch of peptide bonds precisely regulated by prolyl *cis-trans* isomerization plays a central role in many aspects of cellular processes (Lu et al., 2007; Marsolier et al., 2015).

Proline residues in proteins have *cis* and *trans* peptide bond conformations, which are tightly orchestrated by prolyl *cis-trans* isomerization (Lummis et al., 2005; Zosel et al., 2018). Proline conversion occurs very slowly in aqueous solution (Fischer and Aumuller, 2003). But in the presence of peptidyl prolyl *cis-trans* isomerases (PPIases), the *cis-trans* rotation of peptide bond is stimulated, thereby adjusting the spatial arrangement of protein backbone segments (Theuerkorn et al., 2011). There are four evolutionally conserved PPIase subfamilies: cyclophilins, FK506-binding proteins (FKBPs), parvulins, and protein phosphatase 2A phosphatase activator (PTPA) (Thapar, 2015; Zhou and Lu, 2016). Peptidyl-prolyl *cis-trans* isomerase NIMA-interacting 1 (Pin1), a member of parvulins subfamily, was originally identified in 1996 (Lu et al., 1996), and is a unique enzyme that specifically catalyzes the isomerization of phosphorylated serine-proline or phosphorylated threonine-proline (pSer/Thr-Pro) motifs, representing a novel mechanism that protein conformation after Ser/Thr-Pro phosphorylation can be regulated by Pin1 to display alterable biological functions (Lu and Hunter, 2014; Zhou and Lu, 2016). Furthermore, the data from global mass spectrometry analysis have suggested a high percentage of serine/threonine phosphorylation in all phosphorylated proteins (Shi, 2009). Thus, Pin1 is of great interest to scientists committing to the research of molecular cell biology.

Emerging evidence has demonstrated that Pin1-mediated prolyl isomerization exerts a pivotal effect on multiple physiological processes including cell growth, cell cycle regulation, immune response, neuronal differentiation, and tumorigenesis (Sacktor, 2010; Tun-Kyi et al., 2011; Daza-Martin et al., 2019). In cancer—one of the leading causes of human death worldwide (Bray et al., 2018)—Pin1 is widely overexpressed and/or overactivated compared with normal cells or tissues (Pang et al., 2004; Pulikkan et al., 2010; Lu and Hunter, 2014). A high level of Pin1 overexpression/overactivation closely correlates to poor clinical prognosis of diverse cancers (Wang et al., 2015; Zhou and Lu, 2016). Through multiple regulatory mechanisms, Pin1 promotes tumor initiation, development, and drug resistance by acting as an activator of some oncogenes and growth enhancers, or as an inactivator of some tumor suppressors and growth inhibitors (Yeh and Means, 2007; Lu and Hunter, 2014; Zhou and Lu, 2016). Therefore, these achievements provide strong evidence that Pin1 is an attractive target for cancer therapy, leading to the discovery of Pin1 inhibitors for treating cancer and preventing drug resistance.

Given the critical role of Pin1 in cancer, here we review the recent findings about dysregulation, mechanisms, and biological functions of Pin1 in cancer cells, and also discuss the significance and potential applications of Pin1 dysregulation in human cancer.

## Pin1 Dysregulation in Cancer

The *PIN1* gene is located on chromosome 19p13.2 and encodes Pin1 isomerase, composed of 163 amino acids (Lu et al., 1996; Ranganathan et al., 1997; Modena et al., 2006). In normal tissues and cells, the level of Pin1 expression is usually closely correlated to the cell proliferation potential (Saegusa et al., 2010), and Pin1 level in tissues decreases with aging (Lee et al., 2011b). However, Pin1 is aberrantly upregulated or overactivated in many tumors or cells with a tendency to differentiate into tumors (Chen et al., 2018). Varied transcriptional, translational, and post-translational factors contribute to Pin1 dysregulation in cancer cells (Table 1).

**TABLE 1:** Selected factors contribute to Pin1 dysregulation in cancer.

Regulators	Acting sites	Regulatory activity to Pin1 and cancer	Cancer types	References
**Transcriptional regulators**				
E2F	PIN1 promoter E2F site	Activation	Breast cancer	Ryo et al., 2002
C/EBPα-p30	PIN1 promoter E2F site	Activation	Leukemia	Pulikkan et al., 2010
Ras	PIN1 promoter E2F site	Activation	Breast cancer	Ryo et al., 2002; Wulf et al., 2004
c-Neu	PIN1 promoter E2F site	Activation	Breast cancer	Ryo et al., 2002; Wulf et al., 2004
Notch1	PIN1 promoter BS1 site	Activation	Breast cancer	Takahashi et al., 2007; Rustighi et al., 2009, 2013
**Translational regulators**				
miR-200c	3′-UTR of Pin1 mRNA	Inhibition	Breast cancer	Luo et al., 2014
miR-140-5p	3′-UTR of Pin1 mRNA	Inhibition	Hepatocellular carcinoma	Yan et al., 2017
miR-200b	3′-UTR of Pin1 mRNA	Inhibition	Breast cancer	Zhang et al., 2013
miR-296-5p	3′-UTR of Pin1 mRNA	Inhibition	Prostatic cancer	Lee et al., 2014
miR-874-3p	3′-UTR of Pin1 mRNA	Inhibition	Hepatocellular carcinoma	Leong et al., 2017
**Post-translational regulators**				
PLK1	Ser65 of Pin1 protein	Activation	Cervical cancer	Eckerdt et al., 2005
MLK3	Ser138 of Pin1 protein	Activation	Breast cancer Cervical cancer	Rangasamy et al., 2012
SENP1	Lys6, Lys63 of Pin1 protein	Activation	Breast cancer	Chen et al., 2013
DAPK1	Ser71 of Pin1 protein	Inhibition	Cervical cancer	Lee et al., 2011a

Pin1 expression is regulated by a series of transcriptional factors. The E2F family are highly active in nearly all cancer types, regulating gene expression driven by cyclin-dependent kinase (CDK)-Rb-E2F axis (Dick et al., 2018; Kent and Leone, 2019). *PIN1* transcription is stimulated by the E2F family, which is located on the E2F binding sites of the *PIN1* promoter (Ryo et al., 2002). Additionally, E2Fs-mediated Pin1 transcription is also activated by other transcriptional factors. C/EBPα-p30, a mutant of transcription factor C/EBPα, which was found in around 9% of acute myeloid leukemia (AML) patients, induces Pin1 expression by recruiting E2F1 in the *PIN1* promoter and enhances leukemia (Pulikkan et al., 2010). *PIN1* promoter activity is also induced by Neu and Ras signaling via E2F activation in breast cancer (Ryo et al., 2002; Wulf et al., 2004). Unlike other transcriptional factors, Notch1 specifically binds the distal BS1 element of *PIN1* promoter and directly triggers *PIN1* transcription, where Pin1 potentiates Notch1 cleavage by γ-secretase to increase Notch1 transcriptional activity, thereby generating a positive loop to upregulate Pin1 expression in human breast cancer (Takahashi et al., 2007; Rustighi et al., 2009, 2013). Because transcriptional factors of Pin1 are generally overactivated by upstream oncogenic signaling (Pabst et al., 2001; Giuli et al., 2019; Kent and Leone, 2019), the above-mentioned evidence gives an explanation, at least in part, for the upregulation of Pin1 in cancer cells.

Along with transcriptional regulation, Pin1 expression is also controlled at post-transcriptional levels, including mRNA stability and protein translation. miRNAs are a class of small non-coding RNAs that regulate gene expression by repressing protein translation or destabilizing target mRNAs by forming a functional RNA-induced silencing complex (RISC) (Garzon et al., 2010; Inui et al., 2010). Diverse miRNAs are found to regulate Pin1 expression. For example, miR-200c is reported to directly target the 3′-UTR of Pin1 mRNA, thus decreasing Pin1 level in breast cancer (Luo et al., 2014). MiR-140-5p is also identified as a potential negative regulator of Pin1 expression by directly binding to the 3′-UTR of Pin1 mRNA, inhibiting Pin1 translation in hepatocellular carcinoma (Yan et al., 2017). Moreover, miR-200b, miR-296-5p, and miR-874-3p were found to be Pin1-targeted miRNAs (Zhang et al., 2013; Lee et al., 2014; Leong et al., 2017). Given the fact that global miRNA expression is downregulated in tumors (Lu et al., 2005; Hermeking, 2012; Zhang et al., 2015), this reduced miRNA expression could lead to Pin1 overexpression in cancer.

Post-translational regulation is another strategy affecting Pin1 dysregulation. PLK1, a trigger for G2/M transition, mediates phosphorylation of Ser65 in Pin1, stabilizing Pin1 by inhibiting its ubiquitination in human cancer cells (Eckerdt et al., 2005). MLK3, a MAP3K family member, phosphorylates Pin1 on a Ser138 site to activate its catalytic function and nuclear translocation, driving the cell cycle and promoting cyclin D1 stability and centrosome amplification of cancer cells (Rangasamy et al., 2012). By contrast, DAPK1, a known tumor suppressor, associates with and phosphorylates Pin1 on Ser71, which suppresses Pin1 nuclear localization and sustains cell cycle by activating cyclin D1 promoter in cells (Lee et al., 2011a). In addition, SENP1 binds to and deSUMOylates Pin1, leading to increased Pin1 stability and enhanced centrosome amplification and cell transformation during tumorigenesis (Chen et al., 2013). Collectively, Pin1 is aberrantly overexpressed/overactivated in multiple tumors through transcriptional, post-transcriptional, and post-translational regulations.

## Pin1 Participates in Tumorigenesis Via Multiple Mechanisms

Pin1 is mainly localized in the nucleus of both normal and cancer cells, colocalizing with a series of nucleoproteins, such as NEK6 (Chen et al., 2006), but its nuclear-cytoplasmic distribution could be changed upon phosphorylation by kinases including the above-mentioned DAPK1 and MLK3 (Lee et al., 2011a; Rangasamy et al., 2012). Recently, Chen et al. (2018) reviewed 81 Pin1 targets in human cancer. We have checked these targets based on published articles and found that Pin1 regulates 29 targets in the nucleus and 35 targets in the cytoplasm (the rest are unknown for their cellular localization), indicating that Pin1 has no apparent preference between its nuclear or cytoplasmic clients. Additionally, Pin1 participates in cancer development via transcriptional, post-transcriptional, and post-translational mechanisms, and these mechanisms operate in both the nucleus and cytoplasm (Lu and Hunter, 2014; Zhou and Lu, 2016). Thus, Pin1 has both nuclear and cytoplasmic functions, and is extensively involved in the initiation and progression of cancer.

Structurally, Pin1 contains an N-terminal WW domain and a C-terminal PPIase domain, and these two domains are connected by a flexible sequence (Yaffe et al., 1997). It is well-established that WW domain is responsible for specifically recognizing and binding the pSer/Thr-Pro segment of its substrates (Lu et al., 1999; Verdecia et al., 2000), while PPIase domain is the *bona fide* component catalyzing the conformation change of pSer/Thr-Pro’s peptide bond (Yaffe et al., 1997; Lu et al., 2007). Recently, a new opinion has emerged that the WW domain is also an allosteric effector. Substrate binding to Pin1 WW domain changes the intra/inter domain mobility under a stereoselective manner, thereby altering the binding and catalysis in the distal PPIase domain (Namanja et al., 2011; Peng, 2015). The data from computational calculations also support this opinion and further predicts that Ile28 at the flexible sequence between the PPIase and WW domains is a potential key residue responsible for bridging the communication between the two domains to realize Pin1 allostery (Barman and Hamelberg, 2016; Momin et al., 2018). Considering the phosphorylated state of its substrates, Pin1 renders a functional diversity and/or pathological consequences of given substrates (Zhou and Lu, 2016; Chen et al., 2018), which is achieved mainly through three mechanisms: regulating biological activity, protein degradation, and nucleus-cytoplasm distribution of its substrates (Table 2).

**TABLE 2:** Regulatory mechanism of Pin1 in cancer.

Substrates	Motif	Phenotype	Cancer types	References
**Regulating biological activity of Pin1 substrates**				
RNAP II	Ser2-Pro	Regulates cell cycle	Cervical cancer	Kops et al., 2002; Xu and Manley, 2007
BRCA1-BARD1	Ser114-Pro (BRCA1)	Promotes replication fork protection	Osteosarcoma Cervical cancer	Daza-Martin et al., 2019
B-Myb	Not available	Regulates cell cycle	Cervical cancer	Werwein et al., 2019
FAK	Ser910-Pro	Promotes cell migration, invasion, and metastasis	Breast cancer Glioblastoma	Zheng et al., 2009
PTP-PEST	Ser571-Pro	Promotes migration, invasion, and metastasis	Glioblastoma	Zheng et al., 2011
ATR	Ser428-Pro	Prevents apoptosis	Lung cancer Colon cancer	Hilton et al., 2015
Rb	Ser608-Pro Ser612-Pro	Regulates cell cycle	Osteosarcoma Lung cancer	Rizzolio et al., 2012 Tong et al., 2015
ERα	Ser118-Pro	Promotes proliferation	Breast cancer	Rajbhandari et al., 2012 Rajbhandari et al., 2015
Smad2/3	Thr179-Pro	Promotes migration and invasion	Prostate cancer	Matsuura et al., 2010
STAT3	Ser727-Pro	Induce EMT	Breast cancer	Lufei et al., 2007
**Affecting protein degradation of Pin1 substrates**				
NF-κB	Thr254-Pro	Promotes migration	Glioblastoma Leukemia Lymphomas	Ryo et al., 2003 Atkinson et al., 2009
Nanog	Ser52-Pro Ser65-Pro	Promotes cancer stem cell traits	Prostate cancer	Moretto-Zita et al., 2010 Zhang et al., 2019
BRD4	Thr205-Pro	Promotes proliferation, migration, and invasion	Gastric cancer	Hu et al., 2017
Fbw7	Thr205-Pro	Promotes proliferation and transformation	Colon cancer	Min et al., 2012; Bhaskaran et al., 2013
CDK10	Thr133-Pro	Induce tamoxifen resistance	Breast cancer	Khanal et al., 2012
ΔNp63	Thr538-Pro	Promotes proliferation	Oral squamous cell carcinoma	Li et al., 2013
c-Myc	Ser62-Pro	Promotes proliferation	Breast cancer	Farrell et al., 2013
PML	Ser403-Pro Ser505-Pro	Promotes proliferation	Breast cancer	Lim et al., 2011
RUNX3	Thr209-Pro Thr212-Pro Thr214-Pro Thr231-Pro	Promotes proliferation	Breast cancer	Nicole Tsang et al., 2013
HIF-1α	Ser641-Pro Ser643-Pro	Promotes angiogenesis	Colon cancer	Han et al., 2016
**Altering nucleus-cytoplasmic distribution of Pin1 substrates**				
PKM2	Ser37-Pro	Promotes Warburg effect and tumor growth	Glioblastoma	Yang et al., 2012; Yang and Lu, 2013
TRIM59	Ser308-Pro	Promotes tumor growth	Glioblastoma	Sang et al., 2019
p53-RS	Ser249-Pro	Regulates cell cycle	Hepatocellular carcinoma	Liao et al., 2017
XPO5	Ser497-Pro	Promotes proliferation, migration, and invasion	Hepatocellular carcinoma	Sun et al., 2016; Li et al., 2018
Cyclin D1	Thr286-Pro	Promotes cell cycle and proliferation	Nasopharyngeal carcinoma	Liou et al., 2002; Xu et al., 2016

### Regulating Biological Activity of Pin1 Substrates

The biological activities of most human proteins are conformationally specific (Papaleo et al., 2016). Pin1-mediated conformational change significantly impacts their functions. The C-terminal domain (CTD) of the RNA polymerase (RNAP) II plays a critical role in pre-mRNA transcription (Bentley, 2014; Jeronimo et al., 2016). Pin1 affects CTD phosphorylation and RNAP II activity during initiation of the transcription cycle, and not during elongation, suggesting the functional role of Pin1 in RNA transcription (Kops et al., 2002; Xu et al., 2003; Xu and Manley, 2007). Pin1 also enhances BRCA1-BARD1 interaction with RAD51, thereby increasing the presence of RAD51 at stalled replication structures and governing replication fork protection during cancer development (Daza-Martin et al., 2019). Moreover, B-Myb phosphorylated by CDK is isomerized by Pin1, enabling PLK1 docking and subsequent PLK1-mediated B-Myb phosphorylation to stimulate transcription of late cell cycle genes (Werwein et al., 2019). In Ras-activated tumor cells, the function of FAK and PTP-PEST are also regulated by Pin1. Pin1 isomerizes both Ser910-phosphorylated FAK and Ser571-phosphorylated PTP-PEST to enhance the interaction between PTP-PEST and FAK, leading to the dephosphorylation of FAK Tyr397 by PTP-PEST and the promotion of migration, invasion, and metastasis of Ras-related tumor cells (Zheng et al., 2009, 2011).

In addition to activating substrate activity, Pin1 is also able to deactivate substrates. ATR, a PI3K-like protein kinase, has an antiapoptotic activity at mitochondria in response to UV-induced DNA damage. In cancer cells, this mitochondrial activity is reduced by Pin1 that catalyzes ATR from *cis*-isomer to *trans*-isomer at the phosphorylated Ser428-Pro motif (Hilton et al., 2015). Moreover, the function of the tumor suppressor Rb is largely regulated by a dynamic balance of phosphorylation and dephosphorylation. Pin1 directly interacts with the spacer domain of Rb protein, and allows the interaction between CDK/cyclin complexes and Rb in mid/late G1, leading to the inactivation of Rb (Rizzolio et al., 2012; Tong et al., 2015). Subsequently, the Pin1-induced Rb inactivation leads to the dissociation of E2F from Rb and increased E2F transcriptional activity, triggering the expression of cell cycle regulatory proteins and promoting cell cycle progression through the G1 checkpoint in cancer cells (Cheng and Tse, 2018).

### Affecting Protein Degradation of Pin1 Substrates

Pin1 has the ability to prevent protein degradation of oncogenes and growth-promoting regulators. For example, Pin1 associates with the pThr254-Pro motif of transcription factor NF-κB p65 subunit, leading to the increased protein stability of p65 and enhanced transcriptional activity of NF-κB in various cancers, including leukemia, lymphomas, and glioblastoma (Ryo et al., 2003; Atkinson et al., 2009). In prostate cancer, tumor suppressor SPOP interacts with Nanog and promotes Nanog poly-ubiquitination and subsequent degradation, but Pin1 functions as an upstream Nanog regulator and impairs its recognition by SPOP, stabilizing Nanog to promote the cancer stem cell traits and tumor progression (Zhang et al., 2019). Moreover, Pin1 directly binds to and isomerizes phosphorylated Thr204-Pro205 motif of BRD4 to enhance its stability by inhibiting its polyubiquitination, promoting BRD4’s interaction with CDK9 and its transcriptional activity. Substitution of BRD4 with Pin1-binding-defective BRD4-T204A mutant reduces BRD4 stability, which attenuates BRD4-mediated gene expression and suppresses cell proliferation, migration, invasion, and tumor formation, suggesting the positive correlation of Pin1 function and BRD4 stability in gastric cancer cells (Hu et al., 2017).

Pin1 could also promote the protein degradation of tumor suppressors and growth-inhibitory regulators. Fbw7 is the substrate recognition component of the E3 ligase complex and is critical for ubiquitylation and degradation of given proteins (Ji et al., 2015). Pin1 interacts with Fbw7 and induces Fbw7 self-ubiquitination and protein degradation by disrupting Fbw7 dimerization, contributing to oncogenesis. By contrast, depletion of Pin1 in cancer cells leads to elevated Fbw7 expression, which subsequently reduces Mcl-1 abundance, sensitizing cancer cells to taxol treatment (Min et al., 2012; Bhaskaran et al., 2013). An inverse correlation between the expression of CDK10 and the degree of tamoxifen resistance suggests CDK10 could be an important determinant of tamoxifen resistance in breast cancer. Pin1 facilitates CDK10 degradation as a result of its interaction with, and subsequent ubiquitination of, CDK10, thereby suggesting that the Pin1-mediated CDK10 ubiquitination is a major regulator of tamoxifen-resistant breast cancer cell growth and survival (Khanal et al., 2012).

### Altering Nucleus-Cytoplasmic Distribution of Pin1 Substrates

Changing the nucleus-cytoplasm distribution is another mechanism of Pin1 function. A typical example is PKM2. Upon the activation of EGFR signaling, Ser37-phosphorylated PKM2 recruits Pin1 for *cis-trans* isomerization and promotes PKM2 binding to importin α5 and translocating to the nucleus, where nuclear PKM2 acts as a coactivator of β-catenin to promotes the Warburg effect and tumorigenesis (Yang et al., 2012; Yang and Lu, 2013). This process is similar to the recently published mechanism of TRIM59 (Sang et al., 2019). In addition, the mechanism underlying the gain-of-function of p53-R249S (p53-RS), a p53 mutant frequently detected in hepatocellular carcinoma, is also mediated by Pin1. In detail, Pin1 isomerizes p53-RS phosphorylated by CDK4 in the G1/S phase and enhances nuclear localization of p53-RS, resulting in a p53-RS-c-Myc interaction and an elevated c-Myc-dependent rDNA transcription key for ribosomal biogenesis, which promotes cell cycle progression and cell growth of hepatocellular carcinoma (Liao et al., 2017).

Recently, we have demonstrated that Pin1 plays an important role in miRNA biogenesis. XPO5-mediated nucleus-to-cytoplasm export of precursor miRNAs (pre-miRNAs) is a post-transcriptional step in the process of miRNA biogenesis (Lin and Gregory, 2015; Peng and Croce, 2016; Wu et al., 2018). Pin1 blocks nucleus-to-cytoplasm export of XPO5 phosphorylated by ERK kinase, decreasing mature miRNA biogenesis in hepatocellular carcinoma (Sun et al., 2016; Li et al., 2018; Pu et al., 2018). Moreover, this impaired miRNA biogenesis in hepatocellular carcinoma could be restored by novel Pin1 inhibitors and their formulations (Pu et al., 2018; Fan et al., 2019; Sun et al., 2019; Zheng et al., 2019), giving new insight into the therapy of liver cancer.

## Significance of Pin1 Dysregulation in Tumor

**FIGURE 1:**
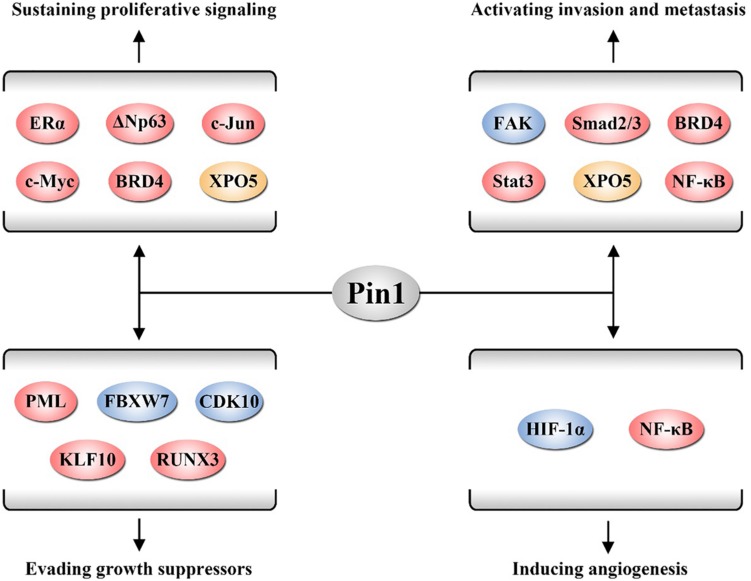
Pin1 is involved in several cancer hallmarks. Pin1 activates a number of oncogenic proteins to sustain proliferative signaling, evade growth suppressors, activate invasion and metastasis, and induce angiogenesis. The proteins displayed in red, yellow, and blue participate in these hallmarks through regulating biological activity, affecting protein degradation, and altering nucleus-cytoplasm distribution of its substrates, respectively.

Following the epoch-making conclusion by Hanahan and Weinberg (2011), the major cancer hallmarks are summarized, such as sustaining proliferative signaling, evading growth suppressors, activating invasion and metastasis, and inducing angiogenesis. Emerging evidence demonstrates that Pin1 promotes cancer by acting as an activator of numerous oncogenes and growth enhancers or as an inactivator of numerous tumor suppressors and growth inhibitors to affect cancer hallmarks (Zhou and Lu, 2016). In this section, we review the roles of Pin1 in these cancer hallmarks (Figure 1).

### Pin1 Sustains the Proliferative Signaling

Cancer cells possess an excessive cell proliferation ability that sustains proliferative signaling (Samatar and Poulikakos, 2014; Bykov et al., 2018). Pin1 is initially identified as a regulator of mitosis and gives rise to sustaining proliferative signaling in multiple cancers.

Cyclin D1, a pivotal cell cycle regulator, promotes cell cycle progression in human cancer (Sherr, 1996). Pin1 interacts with and isomerizes cyclin D1 in a phosphorylation-dependent manner, enhancing the nuclear accumulation of cyclin D1 and triggering cells into cell cycle, and promotes cell proliferation (Liou et al., 2002). Dysregulation of ERα expression also contributes to the proliferation of cancer, especially breast cancer (Brisken, 2013). Pin1 promotes ERα function through several mechanisms. Pin1 isomerizes the Ser118-Pro bond of ERα AF1 region to increase AF1 transcriptional activity, promoting the growth of tamoxifen-resistant breast cancer cells (Rajbhandari et al., 2012). Furthermore, Pin1 can directly regulate the adjacent DNA binding domain of ERα in an allosteric manner, enhancing the DNA binding function of ERα to drive breast cancer proliferation (Rajbhandari et al., 2015).

ΔNp63s, the N-terminal truncated isoforms of p63 lacking the transactivation domain, are associated with human tumorigenesis (Chen et al., 2017). Pin1 interacts with Thr538-Pro of ΔNp63α and disrupts p63α-WWP1 interaction to inhibit the proteasomal degradation mediated by E3 ligase WWP1, promoting ΔNp63α-induced cell proliferation of human oral squamous cell carcinoma (Li et al., 2013). Moreover, Pin1 enhances the stability of BRD4 by inhibiting its ubiquitination and increasing transcriptional activity of BRD4 to promote the proliferation of gastric cancer (Hu et al., 2017). In addition, Pin1 also activates many pro-proliferative proteins to enhance tumor cell proliferation and tumor growth, including c-Myc and XPO5 (Farrell et al., 2013; Li et al., 2018).

### Pin1 Evades Growth Suppressors

There are a number of tumor suppressors that negatively regulate cancer progression within cells, but cancer cells are able to bypass these barriers via various mechanisms. Several works suggest that Pin1 is an expert in injuring tumor suppressors.

The promyelocytic leukemia (PML) is a tumor suppressor involved in apoptosis and DNA damage repair. Pin1 binds and targets PML for degradation in an ERK-dependent manner by targeting Ser403 and Ser505 of PML, inducing the development of breast cancer cells (Lim et al., 2011). Moreover, KLHL20 coordinates with Pin1 and CDK1/2 to mediate hypoxia-induced PML proteasomal degradation, thereby potentiating multiple tumor hypoxia responses in human prostate cancer (Yuan et al., 2011). Furthermore, Pin1 also stabilizes the oncogenic fusion protein PML-RARα, resulting in a decreased anti-proliferative activity of ATRA in AML (Gianni et al., 2009).

Runt-related transcription factor 3 (RUNX3) is an ERα inhibitor in breast cancer (Huang et al., 2012). Pin1 recognizes four phosphorylated Ser/Thr-Pro motifs in RUNX3 via its WW domain to suppress the transcriptional activity of RUNX3 and induce the ubiquitination and proteasomal degradation of RUNX3 in breast cancer (Nicole Tsang et al., 2013). KLF10 is a member of the Krüppel-like transcription factor family and acts as a tumor suppressor, mimicking the anti-proliferative effect of TGF-β in various cancer cells. Pin1 interacts with KLF10 and promotes its protein degradation, blocking the anti-proliferative function of KLF10 in cancer cells (Hwang et al., 2013). Pin1 also interacts with Fbw7 and CDK10 in a phosphorylation-dependent manner and promotes their ubiquitination and degradation, which suppresses their function to trigger cell proliferation and transformation of cancer cells (Khanal et al., 2012; Min et al., 2012).

### Pin1 Activates Invasion and Metastasis

Invasion and metastasis are the leading causes of death in cancer patients and remain the greatest challenges in the clinical management of cancer (Lambert et al., 2017). Mounting works have demonstrated the invasion- and metastasis-promoting function of Pin1 in human cancer.

The transforming growth factor β (TGF-β) signaling pathway is a key player in tumor development, modulating processes including cell motility, where Smad proteins are major downstream effectors of TGF-β signaling (Lamouille et al., 2014). Phosphorylated Thr179-Pro motif of Smad2/3 interacts with Pin1 in a TGF-β-dependent manner, inducing migration and invasion via N-cadherin in prostate cancer cells (Matsuura et al., 2010). In turn, Pin1–Smad3 interaction is reduced by the inhibition of CDK-mediated Smad3 phosphorylation, leading to the suppression of triple negative breast cancer cells (Thomas et al., 2017).

Ras and STAT3 signaling has a significant impact on tumor metastasis. Pin1 binding and prolyl isomerizing of FAK cause PTP-PEST to interact with and dephosphorylate FAK Tyr397, promoting Ras-induced cell migration, invasion, and metastasis of numerous cancers (Zheng et al., 2009, 2011). Pin1 associates with STAT3 upon cytokine/growth factor stimulation to promote STAT3 transcriptional activity and target gene expression as well as recruit transcription coactivator p300, inducing epithelial–mesenchymal transition of MCF-7 cells (Lufei et al., 2007). Additionally, Pin1 enhances the invasion and metastasis of multiple cancers by activating NF-κB, BRD4, and XPO5 (Hu et al., 2017; Li et al., 2018; Nakada et al., 2019).

### Pin1 Induces Angiogenesis

Solid tumors rely on angiogenesis to supply sufficient nutrients and oxygen as well as to eliminate metabolic waste and carbon dioxide for rapidly expanded cancer cells (Chung et al., 2010). The angiogenesis is strictly controlled *in vivo*. Increasing evidence has illustrated that Pin1 is involved in cancer-associated angiogenesis.

Hypoxia-inducible factor 1α (HIF-1α) is responsible for promoting the expression of many genes involved in angiogenesis (Rosmorduc and Housset, 2010). Pin1 directly interacts with HIF-1α at both exogenous and endogenous levels to stabilize the HIF-1α protein in human colon cancer cells and upregulating expression of VEGF, a major contributor to angiogenesis (Han et al., 2016). Moreover, Pin1 cooperates with KLHL20 to induce the ubiquitin-dependent degradation of PML, an inhibitor of HIF-1α-induced angiogenesis, resulting in the activation of angiogenesis in many cancers (Yuan et al., 2011). Additionally, NF-κB is also triggered by Pin1 to promote angiogenesis in hepatocellular carcinoma (Shinoda et al., 2015). By contrast, inhibition of Pin1, through RNAi or small molecular inhibitors, significantly reduces the cancer-induced angiogenesis (Ryo et al., 2005; Kim et al., 2012), further supporting the crucial role of Pin1 in angiogenesis.

## Conclusion

Pin1 is identified as a unique enzyme mediating the *cis-trans* isomerization of pSer/Thr-Pro motif of proteins specifically, extensively participating in the initiation and progression of many human cancers. In this article, we reviewed the existing works on the dysregulation, biological function, molecular mechanism, and significance of Pin1 in cancer cells. These works commonly report that Pin1 is an excellent target for the diagnosis and therapy of diverse cancers. Over the past two decades, diverse small-molecule Pin1 inhibitors were developed and some of them, such as ATRA, KPT-6566, arsenic trioxide, and API-1, exhibited attractive *in vitro* and *in vivo* activity toward human cancer, including acute PML, breast cancer, and hepatocellular carcinoma (Wei et al., 2015; Campaner et al., 2017; Kozono et al., 2018; Pu et al., 2018). However, to date, no Pin1 inhibitors are submitted to clinical trial for cancer treatment. Moreover, Pin1 is also not applied in clinical cancer diagnosis, even though Pin1 seems to be a potential cancer-specific biomarker. Therefore, more effort should be made to fill these gaps.

Despite these efforts, a number of highly relevant questions remain unanswered. First, Pin1 enrichment is precisely orchestrated by multiple regulatory mechanisms. However, the theory about the epigenetic regulation and protein decay of Pin1 is rarely studied. So, the origin of Pin1 dysregulation is not fully understood. Second, mounting data have indicated that non-coding RNAs, especially regulatory non-coding RNAs including miRNA, long non-coding RNA (lncRNA), and circular RNA (circRNA), construct a complex molecular network along with numerous functional proteins to regulate cellular processes as well as canceration (Anastasiadou et al., 2018). But the relationship of Pin1 and non-coding RNAs is still unclear. Third, post-translational modifications, such as phosphorylation, acylation, sumoylation, and glycosylation, could positively or negatively change protein activity without altering the sequences of proteins (Han et al., 2018). However, little is known on how the post-translational modifications modulate Pin1 function. We expect that the answers to these questions will be found in the coming years, pushing Pin1 toward a truly clinical application.

## Author Contributions

WP, YZ, and YP wrote the manuscript, performed revisions, and read and approved the submitted version.

## Conflict of Interest

The authors declare that the research was conducted in the absence of any commercial or financial relationships that could be construed as a potential conflict of interest.
